# Factors Affecting Upper Limb Fracture Opioid Requirements

**DOI:** 10.7759/cureus.56499

**Published:** 2024-03-19

**Authors:** James Zhang, Florence Bradshaw, Michal Duchniewicz, Fernanda W Fernandes, Rahul Geetala, Matjia Krkovic

**Affiliations:** 1 Department of Orthopedics, Addenbrooke’s Hospital, Cambridge University Hospitals NHS Foundation Trust, Cambridge, GBR; 2 School of Clinical Medicine, University of Cambridge, Cambridge, GBR; 3 Department of Trauma, Addenbrooke’s Hospital, Cambridge University Hospitals NHS Foundation Trust, Cambridge, GBR; 4 Department of Surgery, University College Hospital, London, GBR; 5 Department of Trauma and Orthopedics, University of Cambridge, Cambridge, GBR; 6 Department of Trauma and Orthopedics, Addenbrooke’s Hospital, Cambridge University Hospitals NHS Foundation Trust, Cambridge, GBR

**Keywords:** opioids, rehabilitation, major trauma, fracture, upper limb

## Abstract

Introduction

Understanding the different opioid pain relief requirements between patients with upper limb fractures can be useful in forming specific evidence-based guidelines and balancing patient-clinician prescribing discussions with opioid stewardship. We investigated the predictors for opioid requirements in upper limb fractures.

Methods

We retrospectively investigated all upper limb fractures from the shoulder to the wrist treated at a major trauma center from January 2015 to January 2022. The data collected consisted of fracture location, demographics, comorbidities, and management options. Post-injury opioid prescriptions in the first post-injury year were calculated every month up to six months and then grouped from the seventh to the 12th month and converted to morphine milligram equivalents (MMEs). We then calculated days requiring at least one medication (representing the “coverage”) and relative “strength” in each time period.

Results

Six thousand four hundred thirteen patients sustaining a combined 9125 fractures were included in the study, with an MME mean of 436. Fracture locations of the scapula, proximal humerus, humeral shaft, distal humerus, and proximal ulna all had significantly higher MME requirements (p<0.05) at the one-year level. The radius shaft and distal radius had significantly lower MME requirements (p<0.05).

The patients with depression, diabetes, drug abuse history, obesity, pulmonary circulatory disorder, and rheumatological conditions required higher strength of opioids at the one-year level (p<0.05). The patients with chronic kidney disease, depression, pulmonary circulation disorder, and rheumatological conditions required higher coverage of opioids at the one-year level (p<0.05).

Conclusion

Our study presents a high-resolution breakdown of the post-injury opioid requirements for patients with upper limb injuries. Fractures of the scapula, proximal humerus, and shaft of the humerus were associated with increases in both opioid strength and coverage. Depression, pulmonary disease, and rheumatological conditions were all associated with increased opioid strength and coverage. This provides a framework for which clinicians and patients can more accurately anticipate the course of the rehabilitation journey and risk stratify appropriately at the outset of injury.

## Introduction

Approximately 3.7 million surgical procedures are performed in England each year, with 1.2 million being performed under the specialty “trauma and orthopedics” [1]. A large proportion of these surgeries treat and manage fractures. Both fractures and their associated surgeries are painful and require pain relief to ensure patient comfort. For this, opioids are commonly prescribed, especially in the trauma and orthopedics specialty, the third highest opioid prescribers in medicine [2]. Despite providing effective perioperative analgesia, the current literature suggests that pre- and postsurgical opioid prescriptions contribute to the ongoing opioid crisis globally [3]. In addition to opioid abuse, the persistent use of opioids has been associated with an increased risk of cardiac events and bone fracture [4], further contributing to opioid-associated deaths and the costs to healthcare systems [3].

Currently, there are very few opioid prescribing guidelines in orthopedics, and those that exist cover elective procedures [5]; thus, surgeons are mostly prescribing by extrapolating the opioid needs of similar patients. The writing of procedure-specific opioid prescribing guidelines for orthopedic procedures is complex due to the extent of bone and soft tissue injury, the anatomical site involved, the procedure itself, patient comorbidities, and risk factors [6]. Despite the complexity, procedure-specific guidelines could help to solve a small part of the opioid crisis, by tailoring opioid prescriptions to the patient’s needs, countering the abuse and misuse of opioids. As clinicians, we should take accountability for this.

In the literature, there are very few studies focusing on upper limb fracture location and opioid requirements. The patterns that have been described are from the United States [5], and there is no literature in the UK setting. Furthermore, there is little data describing the correlation between different comorbidities and the associated opioid prescription requirements. This study will evaluate the impact of upper limb fracture location and comorbidities on opioid requirements. The results and discussion will inform specific evidence-based guidelines, improve opioid stewardship, and inform patient-clinician prescribing discussions.

## Materials and methods

This study was a retrospective, observational, cohort study investigating the predictive factors for opioid requirements in upper limb fractures in the first post-injury year. All upper limb fractures treated at a major trauma center in the United Kingdom between 1 January 2015 and 31 January 2022 were retrospectively reviewed. Addenbrooke’s Hospital Quality and Safety Information System (QSIS) Local Approval Board issued approval PRN10403. Fractures included all bones from the scapula to the distal radius. Only those with isolated upper limb fractures were included, as the effect of polytrauma fractures can greatly affect opioid requirements that obfuscate the underlying upper limb fracture’s effect on pain levels.

For each patient, the fracture location was documented at the time of admission using International Classification of Diseases (ICD)-10 coding and split into categories of scapula, clavicle, proximal humerus, humeral shaft, distal humerus, proximal ulna, ulna shaft, distal ulna, proximal radius, radial shaft, and distal radius. For patients with multiple distinct fracture locations, multiple codes were used. Each fracture location was further refined into open and closed fractures. For each patient, the following were collected: age, gender, American Society of Anesthesiologists (ASA) grade, and component comorbidities to construct the Charlson Comorbidity Index (CCI), a weighted index of comorbid conditions to gauge mortality likelihood, commonly used in research as a gauge of overall patient comorbidity [7].

All opioid medications in each patient’s first post-injury year were retrieved. Different medication types were then converted into morphine milligram equivalent (MME), defined as the amount, in milligrams, of morphine an opioid dose is equivalent to when prescribed. The most commonly prescribed opioid medication is morphine sulfate, followed by oxycodone hydrochloride. The calculation was done by using the CDC’s opioid conversion factors [8,9]. The start and end date of each medication was used to calculate a distribution of total opioid strength and coverage for the patient over the entire time period.

Python code (Python Software Foundation, Wilmington, DE), a computer program used in data analysis, was then used to convert the raw opioid data into a combined timeline of the rehabilitation period, indicating the overall strength of MME and the number of days in each time period the patient was prescribed at least one opioid medication. The sub-intervals calculated were in one-month blocks until after the sixth post-injury month, and for the final interval, the seventh to 12th post-injury months were combined into one block.

The term “coverage” is defined by the number of days the patient was prescribed at least one opioid within each time period. This indicates the background level of pain the patient experienced that was uncontrolled by non-opioid means, warranting at least one opioid being prescribed. The term “strength” is defined by the total dose of MMEs that the patient was prescribed, from the sum of all opioid medications, over the specific time period. This represents the severity of the overall pain uncontrolled by non-opioid means, as high levels of pain will require higher combined dosage, in addition to widespread coverage.

We first conducted an analysis on operatively treated versus conservatively managed patients across the entire cohort, to elucidate any significant differences between the two groups that would have to be factored into the subsequent analysis.

For location analysis, the open and closed fractures of the same location were grouped into the same category. Then, independent sample t-tests were performed on “strength” and “coverage” variables for the entirety of the first post-injury year and split into individual intervals and compared patients with a certain fracture location with the rest of the cohort.

Finally, comorbidity analysis modelling was performed on the “strength” and “coverage” of opioids using regression analysis, with each sub-interval timeframe being analyzed individually to map out consecutive trends throughout the timeframes. Only those comorbidities whose frequency in the cohort was over 100 were included in the modelling, as tests analyzing smaller-sample-sized comorbidities often were not powered high enough to detect differences. This cutoff was also to highlight the most common comorbidities in the patient population’s effect on opioid usage, as well as to avoid overloading information.

All data analysis was performed with the Statistical Package for Social Sciences (SPSS) version 28.1 (IBM SPSS Statistics, Armonk, NY). Statistical significance was set at p<0.05, with t-tests not assuming equal variance between groups and two-tailed tests being performed to detect any differences.

## Results

A total of 6413 patients had a total of 9125 distinct locations of fracture. The location and demographic breakdowns are shown in Table 1. Fractures of the shaft of the ulna and radius were associated with younger patients (24.22 and 26.26 years, respectively), with the distal humerus having the next lowest average age (34.7 years). There is quite a substantially higher average age for other fractures of the upper limb. These observations are generally reflected in ASA grade at presentation as well, with more proximal fractures of the clavicle and scapula being associated with the highest grades (2.93 and 3.00, respectively), as well as CCI scoring showing lowest values for forearm shaft fractures (0.30 and 0.40 for radial and ulnar shaft, respectively).

**Table 1:** Demographic breakdown of the entire cohort. The data has been sorted by location of the fracture and represented as the number of patients, mean age±SD (years), ASA grade mean±SD, CCI mean±SD, and the number of male and female patients. ASA, American Association of Anesthetists; CCI, Charlson Comorbidity Index; SD, standard deviation

Location of fracture	Number of patients	Age mean±SD (years)	ASA grade mean±SD	CCI mean±SD	Number of male	Number of female
Clavicle	277	51.39±20.12	2.93±1.01	1.47±1.82	487	168
Scapula	655	53.22±22.05	3.00±1.16	1.82±2.40	159	68
Proximal humerus	1133	69.98±19.72	2.62±0.98	3.40±2.37	419	714
Shaft of the humerus	353	52.69±25.13	2.88±1.16	2.00±2.46	201	152
Distal humerus	853	34.70±29.22	2.88±1.06	1.16±2.02	431	422
Proximal radius	436	47.07±24.79	1.94±1.04	1.41±2.03	236	200
Shaft of the radius	522	24.22±19.89	2.15±1.05	0.30±0.92	350	172
Distal radius	2514	49.24±27.93	1.78±1.01	1.77±2.26	1147	1367
Proximal ulna	897	48.88±24.46	2.11±1.01	1.47±1.90	471	426
Shaft of the ulna	547	26.36±21.32	1.84±1.07	0.40±1.10	352	195
Distal ulna	938	41.69±29.61	2.06±1.12	1.41±2.19	506	432

The statistical comparison of operatively treated with non-operatively treated fractures for the entire cohort is shown in Table 2.

**Table 2:** Comparison of non-operatively treated with operatively treated patients in the cohort for both coverage (days) and strength (MME). The data has been sorted by non-operatively treated and operatively treated patients and represented by coverage (in days±SD) and strength (in MME±SD) for each time interval. *Highlighting significance at p<0.05 MME, morphine milligram equivalent; SD, standard deviation

Factor	Non-operatively treated patients	Operatively treated patients	P value
Number of patients	2528	3870	-
1st post-injury month coverage (mean days±SD)	8.86±11.59	8.87±10.23	0.97
2nd post-injury month coverage (mean days±SD)	4.78±10.61	5.01±10.67	0.40
3rd post-injury month coverage (mean days±SD)	3.64±9.60	3.64±9.56	1.00
4th post-injury month coverage (mean days±SD)	3.11±9.05	3.16±9.05	0.84
5th post-injury month coverage (mean days±SD)	2.82±8.69	2.82±8.66	0.98
6th post-injury month coverage (mean days±SD)	2.63±8.43	2.40±8.02	0.28
7th-12th post-injury month coverage (mean days±SD)	33.08±165.88	27.46±145.82	0.15
1st post-injury year coverage (mean days±SD)	58.92±201.09	53.36±179.05	0.25
1st post-injury month strength (mean MME±SD)	150.83±311.96	171.55±341.82	0.01*
2nd post-injury month strength (mean MME±SD)	68.26±202.59	72.79±224.94	0.41
3rd post-injury month strength (mean MME±SD)	48.33±153.49	48.82±172.33	0.91
4th post-injury month strength (mean MME±SD)	40.46±137.76	42.24±160.54	0.65
5th post-injury month strength (mean MME±SD)	36.67±132.07	34.81±135.65	0.59
6th post-injury month strength (mean MME±SD)	34.22±128.23	28.09±117.14	0.05
7th-12th post-injury month strength (mean MME±SD)	52.33±251.71	40.83±216.85	0.05
1st post-injury year strength (mean MME±SD)	431.10±1086.52	439.14±1045.16	0.77

Apart from the first post-injury month for the outcome of strength, there were no statistically significant differences in any timeframe for either strength or coverage between the two groups. This analysis was conducted to elucidate any significance in pain requirement differences between the two groups, which should be factored into further analysis.

The analysis of opioid coverage compared by fracture location (Table 3) reveals strong consecutive sub-intervals of higher opioid coverage requirement for the scapula, proximal humerus, and shaft of the humerus. However, shaft and distal radius fractures showed the strongest consecutive sub-interval and overall trends of lower coverage requirement. Opioid strength showed a stronger association overall with the location of fracture (Table 3), with scapula, proximal humerus, shaft of the humerus, distal humerus, and proximal ulna fractures all showing significantly higher strength requirement over the first year, with only distal radius fractures showing significantly less strength requirements over the year.

**Table 3:** Fracture location association with overall opioid coverage (days) and strength (MME) for the first post-injury year. The data has been sorted by fracture location and represented by coverage (in days±SD) and strength (in MME±SD) for each time interval. *Significantly higher than the average outcome, p<0.05 †Significantly lower than the average outcome, p<0.05 MME, morphine milligram equivalent; SD, standard deviation

Location	Number of patients		Opioid coverage for the first post-injury year overall		Opioid strength for the first post-injury year overall
Scapula	655	Mean (days±SD)	74.54±201.72	Mean (MME±SD)	640.04±1206.58
		Ratio	1.34	Ratio	1.47
		P value	0.01*	P value	<0.01*
Clavicle	277	Mean (days±SD)	49.14±147.28	Mean (MME±SD)	461.41±147.28
		Ratio	0.88	Ratio	1.06
		P value	0.51	P value	0.71
Proximal humerus	1133	Mean (days±SD)	92.20±230.66	Mean (MME±SD)	677.42±1256.51
		Ratio	1.66	Ratio	1.55
		P value	<0.01*	P value	<0.01*
Shaft of the humerus	353	Mean (days±SD)	90.02±209.65	Mean (MME±SD)	788.58±1358.65
		Ratio	1.62	Ratio	1.81
		P value	0.01*	P value	<0.01*
Distal humerus	853	Mean (days±SD)	51.47±150.62	Mean (MME±SD)	542.71±1233.19
		Ratio	0.93	Ratio	1.25
		P value	0.41	P value	0.01*
Proximal radius	436	Mean (days±SD)	38.53±128.72	Mean (MME±SD)	455.76±1275.35
		Ratio	0.69	Ratio	1.05
		P value	0.01†	P value	0.73
Shaft of the radius	522	Mean (days±SD)	29.66±98.80	Mean (MME±SD)	350.74±989.52
		Ratio	0.53	Ratio	0.80
		P value	<0.01†	P value	0.04†
Distal radius	2514	Mean (days±SD)	49.23±186.24	Mean (MME±SD)	374.60±1016.77
		Ratio	0.89	Ratio	0.86
		P value	0.03†	P value	<0.01†
Proximal ulna	897	Mean (days±SD)	54.44±174.83	Mean (MME±SD)	537.41±1289.36
		Ratio	0.98	Ratio	1.23
		P value	0.84	P value	0.01*
Shaft of the ulna	547	Mean (days±SD)	37.84±116.81	Mean (MME±SD)	413.45±1097.16
		Ratio	0.68	Ratio	0.95
		P value	<0.01†	P value	0.62
Distal ulna	938	Mean (days±SD)	50.12±173.22	Mean (MME±SD)	500.16±1178.59
		Ratio	0.90	Ratio	1.15
		P value	0.31	P value	0.07

When looking at the comorbidity association with overall opioid coverage (Table 4), chronic kidney disease, depression, pulmonary disease, and rheumatological conditions all showed statistically significantly positive associations with increased opioid coverage requirements in the first year overall. They also displayed consecutive sub-interval significant associations in the first half year. Obesity, while highly significant over each individual month up until the sixth, did not show significance at the entire one-year level. For opioid requirement strength (Table 4), drug abuse history, obesity, and pulmonary disease all showed significance at every single sub-interval, as well as overall in the entire year, with depression and diabetes showing significance in each of the first three post-injury months and, overall, in the year. Rheumatological conditions showed a strong association only for the first two months; however, this was still overall significantly positively associated with a higher strength of opioid requirement.

**Table 4:** Comorbidity association with overall opioid coverage (days) and strength (MME) over the first post-injury year. The data has been sorted by comorbidity and represented by coverage (in days±SD) and strength (in MME±SD) for each time interval. ​​*Significantly higher than the average outcome, p<0.05 MME, morphine milligram equivalent; SD, standard deviation; CHD, chronic heart disease; CKD, chronic kidney disease

Comorbidity	Number of patients		Opioid coverage for the first post-injury year overall		Opioid strength for the first post-injury year overall
Alcohol	197	Mean (days±SD)	89.15±239.20	Mean (MME±SD)	607.89±1106.12
		Ratio	1.60	Ratio	1.39
		P value	0.09	P value	0.80
CHD	143	Mean (days±SD)	77.28±224.62	Mean (MME±SD)	739.08±1559.91
		Ratio	1.39	Ratio	1.70
		P value	0.79	P value	0.09
CKD	282	Mean (days±SD)	88.64±228.04	Mean (MME±SD)	623.33±1193.64
		Ratio	1.60	Ratio	1.43
		P value	0.01*	P value	0.07
Depression	322	Mean (days±SD)	89.67±224.79	Mean (MME±SD)	701.97±1216.82
		Ratio	1.61	Ratio	1.61
		P value	0.02*	P value	0.01*
Diabetes	470	Mean (days±SD)	68.87±168.44	Mean (MME±SD)	638.66±1083.73
		Ratio	1.24	Ratio	1.47
		P value	0.66	P value	0.01*
Drug abuse	637	Mean (days±SD)	74.48±222.43	Mean (MME±SD)	769.85±1648.60
		Ratio	1.34	Ratio	1.77
		P value	0.09	P value	<0.01*
Liver disease	130	Mean (days±SD)	61.74±145.77	Mean (MME±SD)	618.51±1352.02
		Ratio	1.11	Ratio	1.42
		P value	0.43	P value	0.79
Obesity	113	Mean (days±SD)	97.33±216.91	Mean (MME±SD)	1026.47±1734.47
		Ratio	1.75	Ratio	2.35
		P value	0.07	P value	<0.01*
Pulmonary circulatory disorder	177	Mean (days±SD)	100.44±222.22	Mean (MME±SD)	1013.11±1701.91
		Ratio	1.81	Ratio	2.32
		P value	<0.01*	P value	<0.01*
Rheumatological condition	125	Mean (days±SD)	97.36±228.26	Mean (MME±SD)	784.14±1442.22
		Ratio	1.75	Ratio	1.80
		P value	0.03*	P value	<0.01*

## Discussion

Optimal pain management for fractures is important for both patient well-being and bone healing. Opioids are commonly prescribed to manage this pain. However, the use of opioids comes with risks such as opioid abuse and an increased risk of cardiac events in the long term [4]. Currently, there are very limited data and prescribing guidelines surrounding the prescription of opioids for upper limb fractures and associated surgery [10].

The guidelines that do exist for opioid prescribing are based on chronic pain conditions and not fractures and therefore are not specific [11]. In a study that gathered data on opioid prescription (measured in number of pills rather than MME) prescribed for total knee replacement, all patients were prescribed 30 pills as standard, but on average, only 11 pills were taken. This leaves 19 pills unused suggesting overprescription [12], thus calling for more comprehensive guidelines in orthopedic pain management.

Hsu et al. aimed to produce comprehensive guidelines and recommendations on the management of pain that can be used by orthopedic practices [11]. Hsu et al. gathered a panel of 15 people with expertise in orthopedic trauma and pain management. In addition to recommending alternative management strategies to opioids for pain management, they also recommended basic guidelines for opioid prescription in musculoskeletal injury or surgery. Despite providing some recommendations, mainly “the prescriber should use the lowest effective dose for the shortest period possible” [11], the guidance is very nonspecific and does not identify factors that could mean that a patient requires less or more pain relief. Trauma to the upper limb is diverse in terms of the mechanism of injury, patient demographics (including comorbidities), location of the injury, and associated procedures; thus, it is important to understand how these factors will influence opioid prescription to manage pain [13].

In this retrospective, observational, cohort study, we compare the fracture location and patient comorbidities to opioid coverage and strength, highlighting fracture locations and comorbidities with increased opioid requirements. Identifying these factors will help inform the writing of opioid prescribing guidelines for upper limb fractures.

Fracture location and opioid prescribing

Fractures to the scapula, proximal humerus, and shaft of the humerus are associated with significantly higher opioid coverage over the first post-injury year (74.5, 92.2, and 90.0 days, respectively), whereas the shaft of the ulnar and proximal, shaft, and distal radius fractures were associated with lower opioid coverage over the same time period (37.8, 38.5, 29.7, and 49.2 days, respectively). When looking at the individual time intervals for opioid coverage, it can be seen that the scapular and shaft of the humerus fractures require significantly more opioid coverage days in the first six post-injury months, whereas proximal humerus fractures require more opioid coverage throughout the whole post-injury year.

Similar patterns were seen in opioid strength and fracture location with significantly higher opioid strengths required over the first year in the scapula, proximal humerus, shaft of the humerus, distal humerus, and proximal ulna fractures (640, 677, 789, 543, and 537 MME, respectively). The shaft of the radius and distal radius fractures required less opioid strength requirements (351 and 375 MME, respectively). Looking at the individual time intervals, we can see that scapula and proximal humerus fractures require higher strengths of opioids in the first post-injury year, whereas shaft of humerus fractures are only associated with high opioid strengths in the first six months post injury. Proximal ulna fractures were associated with increased opioid strengths for the first, second, fourth, and fifth months. Rather interestingly, distal humerus fractures were only associated with increased opioid strength prescription in the first month, suggesting that these fractures are more painful initially.

Our data shows that the most painful upper limb fracture (as determined by the coverage and strength of opioid needs) is a fracture of the proximal humerus, whereas the least painful is a fracture of the radial shaft. A fracture of the humerus requires more force compared to a fracture of the radius [14], so there is likely to be more associated injury to the muscle and vasculature around the bone [15], thus increasing pain requirements. In addition, the surrounding musculature of the humerus can increase forces over the fracture site, putting stress on the fracture. Attached to the humerus are 13 muscles that involve shoulder, hand, and elbow movements. Thus, the application of load to these muscles will cause pain over the fracture site, thus requiring more pain relief [14]. The humeral shaft, especially, is very closely related to the radial nerve, which lies in the spiral groove of the humerus. The radial nerve is suggested to be damaged in 8%-16% of humeral shaft fractures [16], and this raises to 60% in open humeral shaft fractures [15], which could contribute to the pain experienced in humeral shaft fractures.

There are various studies from the United States that have compared fracture location and opioid prescription in the upper limb. Cunningham et al. investigated the opioid requirements of the clavicle through the distal radius and compared the opioid demand at the one-month preoperative period to the one-year post-injury point in various intervals measured in oxycodone 5 mg equivalents [5]. They found that humeral fractures were associated with greater opioid prescriptions, and the distal radius fractures had the lowest demand. This agrees with our findings that the humeral fractures are the most painful. However, Cunningham et al. found that distal radius fractures required the lowest opioid demand [5]. However, in our results, the distal radius required significantly lower opioid requirements, and the shaft of the radius required the lowest opioid demands.

Another study done in the United States by Bhashyam et al. also compared discharge prescription by anatomical location [10]. Bhashyam et al. [10] and Cunningham et al. [5] agree that fractures of the distal radius are associated with lower opioid demands, but Bhashyam et al. conclude that diaphyseal radius/ulna fractures had the highest opioid requirements [10]. However, this study only looked at discharge prescriptions, and there may be variation in opioid demand in the recovery period after injury, so it is hard to draw direct comparisons of opioid demand and fracture location from Bhashyam et al.’s study [10].

There is very limited data comparing location and opioid requirements for upper limb fractures. However, despite slight variation in anatomical location, both Cunningham et al.’s study [5] and this study agree that there is a trend of increased opioid requirements for more proximal fractures. This observation can be used to inform guidelines on upper limb fracture opioid prescribing.

Comorbidities and opioid prescribing

Depression, pulmonary disease, and rheumatological conditions all required a significantly higher coverage of opioids across the first post-injury year (89.7, 100.4, and 97.4 days, respectively, compared to an average of 55.6 for the entire cohort in the first post-injury year). For all these comorbidities, the associated higher coverage was seen in the first six months after injury. Obesity, although required a significantly higher coverage of opioids for the first six months, was not significant overall for the first post-injury year. Depression, pulmonary disease, and rheumatological conditions were also associated with significantly higher opioid strengths (702.0, 1013.1, and 784.1 MME, respectively, in the first post-injury year), in addition to diabetes, drug abuse, and obesity (638.7, 769.9, and 1026.5 MME, respectively, compared to an average of 436 MME for the entire cohort in the first post-injury year). Diabetes and depression only saw increased opioid strengths in the first three months, whereas drug abuse, obesity, pulmonary disease, and rheumatological conditions were associated with increased opioid strengths each month for the first post-injury year. Liver disease was never associated with an increased coverage or strength of opioid prescription throughout the whole first post-injury year.

Depression, Drug Abuse, and Opioid Requirements

The literature has shown that younger age, depression, and substance abuse have all been associated with increased opioid consumption after fracture. Cunningham et al. analyzed upper limb fractures and opioid prescriptions from one-month pre-injury to one-year post injury (in oxycodone equivalents). They observed that drug abuse was associated with increased pill requirements throughout the whole year post injury, matching our findings [5]. In a later study, Cunningham et al., however, did not find that depression was associated with increased pill requirements [17]. A study by Sun et al. looked at risk factors for increased opioid demands after surgery. The study looked at numerous operations from different specialties, so this cannot be directly applied to fractures [18]. Despite this, Sun et al. identified that if a patient was taking antidepressants before surgery, they are at risk of increased opioid consumption in the period after [18].

Diabetes and Opioid Requirements

Previous literature has shown that there is a correlation between glycosylated hemoglobin (HbA1c) level and post-operative opioid consumption. Opioid consumption is 20% higher in patients with an HbA1c level of >6.5% in the 48 hours after surgery, and they are more likely to experience poor post-operative pain control. Leading on from this, diabetics, in the immediate post-operative or post-injury recovery period, often are hyperglycemic. Hyperglycemia increases the levels of proinflammatory cytokines and increases the level of pain experienced [19]. This could explain our observation of the increased coverage and strength of opioids in the three- and four-month post-injury period, respectively, in those patients with diabetes.

Obesity and Opioid Requirements

There is little in the literature on obesity and opioid requirements after an upper limb fracture. However, there are studies on opioid requirements in obese patients after fracture and subsequent orthopedic procedures, which can help us begin to understand our observations. A study looking at hip fractures and opioid demands after surgery found that there was an increase in opioid demand in obese patients, possibly due to the pro-inflammatory state, which is present in obesity being worsened. This has also been reflected in other studies looking at the relationship between obesity and opioid use in the post-operative period [20]. It has also been shown that increases in BMI correlate with higher opioid requirements after surgery [21]. This agrees with our observation that obese patients require significantly higher opioid requirements. However, despite requiring increased opioids, obese patients have an increased sensitivity and a higher risk for respiratory depression and obstructive breathing, which is a cause for concern when prescribing opioids [22].

Pulmonary Disease and Opioid Requirements

Our study found that pulmonary disease was associated with significantly increased opioid coverage and strength. Previous studies have also made this observation after major surgery, possibly attributing the increase to unrelieved pain conditions, which those with pulmonary disease may experience [4]. Furthermore, those with advanced pulmonary disease may be prescribed very low doses of opioids to help with associated dyspnea. Many previous studies, such as that by Broggi et al., have identified that previous opioid prescription increases the opioid demand in the post-injury period after bone fracture [23] and could partly explain the increased opioid demands. However, the above suggestions and observations do not necessarily agree with the opioid prescribing guidelines that are present. Some opioids are contraindicated in pulmonary disease due to worsening hypercapnia and the potential effects of respiratory depression. Despite this, some opioids, such as tramadol (a synthetic weak opioid), can be used as there are no clinically relevant respiratory effects and prove to be particularly useful in patients with poor lung function [24]. Our study used MME as a way of comparing opioid requirements; thus, it is hard to tell if the increase in opioid demand in respiratory diseases is due to opioids such as tramadol.

Rheumatological Conditions and Opioid Requirements

Opioids are commonly used in the treatment of rheumatological conditions, especially for short-term pain control [25]; hence, those patients with a rheumatological condition are already at risk of opioid use and related harms [26]. At the time of writing, there is nothing in the literature discussing rheumatological conditions and opioid demands after bone fracture. However, the fact that those patients with rheumatological conditions are often prescribed opioids before injury could explain our observation that rheumatological conditions are associated with increased opioid demands in the post-injury period, linking again with the suggestion made by Broggi et al. [23]. It would be interesting to investigate how opioid consumption after an upper limb fracture varies between different rheumatological conditions. It has already been shown that opioid prescription to manage pain associated with rheumatological conditions varies between conditions, with opioid prescriptions being the highest in ankylosing spondylitis [26].

Liver Disease and Opioid Requirements

As opioid metabolism occurs in the liver and liver disease results in reduced hepatic function, the half-life of opiates will increase. This increases the risk of opioid toxicity in those with liver disease. Thus, when prescribing opioids for post-injury pain, the dosage has to be decreased, and the time between doses has to be increased to avoid the accumulation of opioids to toxic levels [27]. This agrees with our findings that in liver disease, there is no significant increase in the coverage or strength of opioid prescription in the first post-injury year.

The findings of this study begin to identify risk factors, which need to be considered when writing opioid prescribing guidelines after an upper limb fracture. However, the data is limited and varies for many of the comorbidities, and we only explored a small proportion of possible comorbidities. It is highly likely that there are other comorbidities that are associated with an increased opioid demand in the post-injury period.

Limitations, further considerations, and future directions

Our study was retrospective, with a large number of patients over a long timeframe, but was based on one hospital in the United Kingdom, making it hard to draw comparisons with the rest of the United Kingdom. We also cannot directly comment on the patient’s opioid consumption due to the retrospective nature of the study, rather only on their prescribed dosage and regime. We only obtained prescription records rather than the amount of opioids that each patient reported they consumed directly. However, the fact that there were prescription records over multiple months can suggest some level of patient opioid consumption. In the literature, similar studies also use the prescription-filled metric as the surrogate for opioid consumption, as it can be very difficult to effectively monitor when patients are discharged into the community.

While this may influence the absolute strength and coverage values for each patient, the trends and associations with fracture location and comorbidity should mostly be preserved, given that there is no reason to assume one subset of patients would be less compliant in taking prescribed medications than another group. Studies in the literature all standardize opioid consumption in a variety of different ways, making it hard to draw comparisons from paper to paper. Some papers report on the number of pills, MME, or oxycodone equivalents. In addition, the conversion rates to standardize the opioid consumption dose vary between sources [9,28]. From the data set, we did exclude those patients prescribed with continuous large-dose opioids, which went on indefinitely with no follow-up, as this could be a prescribing error. Despite this, there were no other obvious systematic problems or biases in the data set or in our results.

We also did not include all the possible comorbidities, leaving a gap in our data. This does provide an area for future research to see if there is a relationship between other comorbidities and the consumption of opioids after an upper limb fracture. In addition, our data did not include the treatment of each fracture. We grouped both open and closed fractures, of which the management can be very different, thus possibly dictating opioid demand in the recovery period. We also did not include other methods of pain relief, such as the use of nonsteroidal anti-inflammatory drugs (NSAIDs), which could lessen the demand for opioid use.

Another limitation of our study can be seen in how the anatomical location of fractures is defined at diagnosis. Our data is based on doctors’ diagnosis of the injury and subsequent clinical coding. Hence, we could not use the AO Foundation/Orthopaedic Trauma Association (AO/OTA) classification, which is normally used in research but is not used in clinical practice.

Further studies should work to see which factors affect opioid requirements in fractures of the hand, pelvis, and lower limb to complete the main “orthopedic” areas of the body. It would be also interesting to see if there is a relationship between the socioeconomic status of the patient and opioid demands after an upper limb fracture. Researching into these areas will provide more evidence for future opioid prescribing guidelines in orthopedic settings. Despite only including patients who had isolated upper limb fractures, we are aware that other injuries such as soft tissue trauma will affect the amount of opioids required. In further studies, we propose a “trauma score” to be used as a marker of other injuries and the baseline level of pain.

Despite this, our data reflects opioid prescribing after an upper limb fracture and has begun to identify anatomical location and comorbidities, which increase the risk of opioid consumption post injury in the UK setting.

## Conclusions

This retrospective study focused on the impact of upper extremity fracture location (from the clavicle to the distal radius) and patient comorbidities on opioid strength (MME) and coverage (days) in the first post-operative year every month up to six months and then from the seventh to the 12th month. Fractures of the scapula, proximal humerus, and shaft of the humerus were associated with increases in both opioid strength and coverage, whereas the shaft of the radius and distal radius required lower opioid coverage and strength. Depression, pulmonary disease, and rheumatological conditions were all associated with increased opioid strength and coverage. Our study begins to construct a large data set to aid evidence-based decisions for clinicians prescribing opioids in upper limb fractures. The identification of these risk factors and the creation of opioid prescription guidelines thus improve opioid stewardship.
